# Information, deliberation, and decisional control preferences for participation in medical decision‐making and its influencing factors among Chinese cancer patients

**DOI:** 10.1111/hex.13312

**Published:** 2021-07-26

**Authors:** Lin Xiao, Meifang Peng, Yawei Liu, Lili Zhang

**Affiliations:** ^1^ Evidence‐based Nursing and Midwifery Practice Center, School of Nursing Southern Medical University Guangzhou China; ^2^ Department of Medical Oncology Affiliated Cancer Hospital and Institute of Guangzhou Medical University Guangzhou Guangdong Province China

**Keywords:** cancer, influencing factors, medical decision‐making, preference

## Abstract

**Background:**

Patient‐centred care should strive to respond to the individual patient's needs and preferences when possible. Yet, preferences of cancer patients for participation in different stages of the medical decision‐making process to increase matching of preferred and actual participation of the patients in decision‐making are not well known.

**Objective:**

This study aimed to determine the level of information, deliberation and decisional control preferences in decision‐making practices among Chinese cancer patients and to explore their association with the sociodemographic and clinical variables of the patients.

**Methods:**

A cross‐sectional study was conducted involving a convenience sample of 328 cancer patients from three public hospitals in Guangzhou, China. The Patient Expectation for Participation in Medical Decision‐making Scale (PEPMDS) was used to evaluate information, deliberation and decisional control preferences of the patients. Binary logistic regressions were conducted to determine the association between sociodemographic variables, clinical variables and preferences of the patients.

**Results:**

Most patients reported a high preference for information (73.2%) and deliberation (73.8%), while a small number (37.2%) reported a high preference for decisional control. Younger patients and patients with higher levels of education were significantly more likely to have a high preference for information, deliberation and decisional control. Patients with low annual family incomes were significantly more likely to have a low preference for decisional control.

**Conclusion:**

Preferences of patients for involvement in different stages of decision‐making practices could vary. The level of preferences appears to be related to the patient's age, education level, and financial status.

**Practice Implications:**

Healthcare providers should tailor the participatory approaches of patients considering individual preferences for information, deliberation and decisional control during medical decision‐making.

**Patient Contribution:**

Patients participated in the survey and filled in the questionnaires.

## 1 INTRODUCTION

Patient involvement in the medical decision‐making process has been considered as a core element in patient‐centred care.^1^ Research has shown benefits of patient involvement in decision‐making practices for patients (e.g., increased satisfaction, improved treatment outcomes and health‐related quality of life) and benefits related to the clinical encounter (e.g., reduced cost and improved quality of care).^2^, ^3^ In the context of cancer, in particular, decision‐making is fraught with uncertainty, and some decisions are very difficult because of their preference‐sensitive nature, and require taking into consideration the values and preferences of the patients. Therefore, decision‐making practices should strive to respond to the preferences of the individual patients for involvement when possible. Yet, previous studies have shown discrepancies between preferred and actual involvement in the treatment decision‐making process among cancer patients^4^ as it has been shown that the preferred and actual roles of cancer patients in the treatment decision‐making practice match in about 35%–65% of the cases.^5^, ^6^, ^7^ Most studies found that patients desired more involvement initially than what actually occurred.^4^ Also, it has recently been recommended that patients should be convinced to act as autonomous decision‐makers^8^ and most cancer patients are highly anxious when forced to participate beyond their expectations. Hopmans and colleagues conclude that we should involve patients more often but also respect their preferences for participation in decision‐making practices.^9^ Individualizing the approach to decision‐making will allow us to maximize the respect to the patients in our care.^10^ As for decision‐making practices, distinguishing preferences of patients in different stages of the medical decision‐making process is an essential step to match their preferred and actual levels of involvement in medical decision‐making.

Although many studies have been conducted to explore the expectation of patients to participate in decision‐making practices, this topic is still underexplored, especially in Mainland China. A narrative review^11^ has shown that previous studies had mainly explored the preferred role of patients in treatment decision‐making and the Control Preferences Scale (CPS) was the most commonly used instrument. Overall, the CPS is an easy‐to‐administer, valid and reliable measure of preferred and actual roles in medical decision‐making among cancer patients.^12^ It consists of five statements to elicit the role preferences of patients in decisional control over treatment decision‐making and classifies the preferred roles of patients as ‘active’, ‘collaborative’ or ‘passive’. However, the preferred roles are not a reliable indication of the preferred level of involvement of patients since they have different preferences for involvement during different stages of the decision‐making process. Evidence has shown that not all stages of shared decision‐making processes are equally desired by patients.^13^ The conceptual framework of shared decision‐making by Charles et al.^14^ is the widely used model in the literature.^15^ It describes the decision‐making process in three analytic stages: information exchange, deliberation and control over the final decision. Although the Autonomy Preference Index (API)^16^ and the Health Opinions Survey (HOS)^17^ were validated instruments that can capture fundamental parts of the shared decision‐making model by Charles et al.,^14^ both cannot measure all stages of the treatment decision‐making process. The API includes two scales to distinguish between information seeking and decisional control, but has no explicit items about deliberation. The HOS includes two subscales to distinguish between preferences for information and behavioural involvement, but does not measure the decisional control. Besides, the Health Information Wants Questionnaire (HIWQ), developed and validated by Xie et al.,^18^, ^19^ was also a widely used instrument, including two parallel scales, measuring preferences for seven specific aspects of health information and for participation in the corresponding types of decision‐making. The content of HIWQ is relatively comprehensive, but it does not measure the preference for deliberation. To fully understand and distinguish patient preferences in decision‐making practices, a validated instrument is essential to measure preferences for involvement in all stages of the decision‐making process as conceptualized by Charles et al.^14^ Guided by the above‐mentioned decision‐making model of Charles et al.^14^ and on the basis of the items of API and HOS, Xu et al.^20^ developed the Patient Expectation for Participation in Medical Decision‐making Scale (PEPMDS) in relation to the Chinese cultural context. The Delphi method was used for the evaluation of items to ensure content validity. Its psychometric test was conducted in a population of 400 inpatients including cancer patients. Item discrimination, response analysis, *t* test, correlation analysis and factor analysis were used to screen items to ensure that the items have good sensitivity, independence and representativeness.^20^ Exploratory factor analysis showed that the PEPMDS consists of three dimensions: information, deliberation and decisional control preferences, which was consistent with the theoretical model of Charles et al.^14^

Discrepancies between the preferred and the actual level of participation in medical decision‐making among cancer patients represent a common clinical phenomenon worldwide. Yet, preferences of cancer patients in terms of involvement in different stages of the decision‐making process are not well known, especially in Mainland China. Besides, a review by Say et al.^11^ demonstrates that various sociodemographic and clinical variables have been found to be related to the preferred roles of patients in decision‐making and some are inconsistent across studies. Still, little is known about this in Mainland China. The processes of patient involvement in decision‐making practices have considerable variability within and among cultures^21^ and are influenced by legal and economic differences. Consequently, little information is available to aid Chinese physicians in increasing matching of the preferred and actual levels of participation of cancer patients in the decision‐making practice. To fill this gap, we conducted this cross‐sectional study to explore the preferences of cancer patients in all stages of the decision‐making process by adopting the Chinese scale 'PEPMDS' and also explored their association with the sociodemographic characteristics and medical variables of the patients. The results of this study will provide details about the preferences of cancer patients for involvement during different stages of the decision‐making process and provide new insights into decision‐making practices from a Chinese perspective.

## 2 METHODS

### 2.1 Study design and participant

This study was designed as a descriptive cross‐sectional study. Cancer patients were consecutively recruited from three public/teaching tertiary hospitals in Guangzhou, China, from May to July 2020. The eligibility criteria were as follows: (1) age ≥ 18 years, (2) diagnosis of either colorectal, breast or lung cancer, (3) able to read and understand the questionnaire, (4) no cognitive impairment or brain metastasis and (5) willing to participate in the study. Participants were excluded from the study if their family members tend to keep patient's condition a secret. Colorectal, breast and lung cancers were chosen as they are among the most common cancers diagnosed in China and worldwide.^22^ Recruitment was undertaken with the help of the nursing staff working there. Data were collected by face‐to‐face and one‐to‐one surveys conducted by three trained postgraduate and undergraduate students.

### 2.2 Measures

Data on information, deliberation and decisional control preferences of the patients as well as the potentially influencing factors related to sociodemographic and clinical characteristics were obtained.

#### 2.2.1 Sociodemographic and clinical variables

Sociodemographic variables, such as age, gender, marital status, educational level, residence and annual family income, were obtained from the respondents using a self‐prepared questionnaire and the information on illness‐related variables, such as cancer type, cancer stage and duration of cancer, was collected from the medical records of the patients.

#### 2.2.2 Patient preference**s** for involvement in the decision‐making process

The Patient Expectation for Participation in Medical Decision‐making Scale (PEPMDS) is a 12‐item self‐reported scale that covers information, deliberation and decisional control preferences of patients. Each item is rated on a 5‐point Likert‐type scale: 1 = *completely disagree*, 2 = *disagree*, 3 = *neutral*, 4 = *agree*, and 5 = *completely agree*. Nine items are worded positively and the other three items are worded negatively, which were reverse‐scored so that all items indicated preferences for more participation in decision‐making practices. The dimension of information preference consists of three items, related to the needs of illness information. The dimension of deliberation consists of six items, related to the needs of sharing treatment information, and expressing and discussing treatment preferences. The dimension of decisional control preference consists of three items, related to the needs of personal control over the decision. The total score of the above three dimensions ranges from 3 to 15, 6 to 30 and 3 to 15, respectively. The average score of the dimension item is more than 3, representing a high level of preference. For dimensions of information and decisional control preferences, a total score ≤9 is considered to indicate low information preference and low decisional control preference, while a total score >9 is considered to indicate high information preference and high decisional control preference. For the dimension of deliberation preference, a total score ≤18 is considered to indicate low deliberation preference, while a total score >18 is considered to indicate high deliberation preference. PEPMDS has good content and construct validity among Chinese patients.^20^ Before our formal investigation, we conducted a pilot study among 30 cancer patients by convenience sampling to test the internal consistency, and good reliability was achieved (Cronbach's *α* = .89).

### 2.3 Statistical analysis

After data were double‐checked during the data input phase, statistical analyses were performed using SPSS for Windows version 23.0, with the level of significance set two‐sided at *p* < .05. Descriptive statistics were used to describe sociodemographic and clinical variables of the respondents and their preferences for involvement in decision‐making practices. The information, deliberation and decisional control preferences were categorized into two separate groups, respectively, and we first performed univariate analyses to test for an individual predictor with a dependent variable. Consequently, the potential predictors were included in the multivariate analysis if the criterion (*p* ≤ .10) was fulfilled. The binary forced‐entry logistic regressions were used to determine the factors predicting information, deliberation and decisional control preferences. The sample size of our study was considered adequate for a logistic regression model of 10–15 times per covariate in binary outcomes according to the rule of thumb.^23^

### 2.4 Ethics statement

At inclusion, all respondents received a cover letter with information about the study purpose and principles of voluntary participation in the study, including a request for informed consent. After participants provided informed consent, each of them was provided a self‐reported questionnaire to fill in. The clinical information was obtained from the medical records with the consent of the hospitals and doctors. Data were anonymized and were only accessible to the researchers. This study was approved by the Institutional Review Boards of Southern Medical University, Guangzhou, China.

## 3 RESULTS

### 3.1 Sociodemographic and clinical characteristics

A total of 335 cancer patients were recruited and 328 (98.0%) fully completed the questionnaires. The sociodemographic and clinical characteristics of the respondents are shown in Table 1. The mean age of the respondents was 49.35 years (*SD* = 13.89, range 18–77). The majority of them were married (85.4%), and over half of the respondents lived in a town or city (53.0%). Patients had the following diagnoses: 51.2% with colorectal cancer (*n* = 168), 36.3% with breast cancer (*n* = 119) and 12.5% with lung cancer (*n* = 41).

**Table 1:** Characteristics of the respondents and their association with information preference (*n* = 328)

	All respondents (*n* = 328)		Low information preference (*n* = 88)	High information preference (*n* = 240)	Univariate analysis		Multivariate analysis	
Characteristics	Range or number	Mean ± *SD* or % or median (P_25_, P_75_)	(Mean ± *SD*) or median (P_25_, P_75_) or *n* (%)	(Mean ± *SD*) or median (P_25_, P_75_) or *n* (%)	Crude *OR* (95% CI)	*p* value	Adjusted *OR* (95% CI)	*p* value
Age (year)	18–77	49.35 ± 13.89	56.81 ± 13.09	46.61 ± 13.17	0.940 (0.919–0.960)	**<.001**	0.947 (0.920–0.976)	**<.001**
Gender								
Female	158	48.2	45 (13.7)	113 (34.5)	Ref			
Male	170	51.8	43 (13.1)	127 (38.7)	1.176 (0.721–1.918)	.515		
Marital status						.121		
Unmarried	30	9.1	5 (1.5)	25 (7.6)	Ref			
Married	280	85.4	75 (23.0)	205 (62.5)	0.547 (0.202–1.480)	.235		
Divorced or widowed	18	5.5	8 (2.4)	10 (3.0)	0.240 (0.076–1.051)	.102		
Education level						**<.001**		**<.001**
Primary school	92	28.0	58 (17.7)	34 (10.4)	Ref		Ref	
Middle school	127	38.7	24 (7.3)	103 (31.4)	7.321 (3.963–13.523)	**<.001**	5.584 (2.806–10.957)	**<.001**
High school or college	65	19.8	3 (0.9)	62 (18.9)	23.314 (6.704–81.077)	**<.001**	16.144 (4.187–62.252)	**<.001**
Bachelor or above	44	13.4	3 (0.9)	41 (12.5)	35.255 (10.268–121.045)	**<.001**	26.048 (7.127–95.202)	**<.001**
Residence								
Rural	154	47.0	55 (16.8)	99 (30.2)	Ref		Ref	
Town or city	174	53.0	33 (10.0)	141 (43.0)	2.374 (1.436–3.923)	**.001**	0.828 (0.408–1.681)	.601
Annual family income						**.005**		.602
<50,000 RMB	154	47.0	54 (16.5)	100 (30.5)	Ref		Ref	
50,000–150,000RMB	143	43.6	30 (9.1)	113 (34.5)	2.034 (1.208–3.425)	**.008**	1.081 (0.543–2.150)	.651
>150,000RMB	31	9.4	4 (1.2)	27 (8.2)	3.645 (1.212–10.961)	**.021**	1.546 (0.417–5.731)	.515
Cancer type						.145		
Breast	119	36.3	35 (10.7)	84 (25.6)	Ref			
Colorectal	168	51.2	15 (4.6)	26 (7.9)	0.722 (0.342–1.526)	.394		
Lung	41	12.5	38 (11.6)	130 (39.6)	1.425 (0.835–2.434)	.194		
Cancer stage						.381		
Stage I	10	3.0	3 (0.9)	7 (2.1)	Ref	**<.001**		**.045**
Stage II	47	14.3	5 (1.5)	42 (12.8)	3.600 (0.689–18.555)	.126	1.277 (0.200–8.136)	.796
Stage III	143	43.6	29 (8.9)	114 (34.8)	1.685 (0.410–6.919)	.469	0.895 (0.183–4.374)	.891
Stage IV	128	39.0	51 (15.5)	77 (23.5)	0.647 (0.160–2.619)	.542	0.400 (0.081–1.696)	.260
Duration of cancer(month)	1~110	4 (2.8)	3 (2.6)	4 (2.9.25)	1.005 (0.987–1.023)	.602		

*Note*: Bold *p* values are statistically significant.

Abbreviations: 95% CI, 95% confidence interval; OR, odds ratio.

### 3.2 Preferences for involvement in medical decision‐making

The overwhelming majority of respondents reported high preferences for information (73.2%, *n* = 240, total score >9) and deliberation (73.8%, *n* = 242, total score >18), while only 37.2% (*n* = 122) reported a high preference for decisional control (total score >9). Concurrence of high preferences for information, deliberation and decisional control was reported by 115 respondents (35.1%) and concurrence of low preferences for information, deliberation and decisional control was reported by 74 respondents (22.6%), while 139 respondents (42.3%) reported inconsistent preferences for information, deliberation and decisional control.

### 3.3 Association between sociodemographic and clinical characteristics of patients and preferences of patients for involvement in medical decision‐making

Univariate analysis demonstrated that age, education level, residence and annual family income were significantly associated with the preferences for information, deliberation and decisional control, respectively (Tables 1, 2, and 3). Younger patients, patients with higher levels of education, patients who lived in a town or city and patients with higher family income were significantly more likely to have high preferences for information, deliberation and decisional control. Besides, cancer stage was also significantly associated with the preferences for information, deliberation and decisional control, but no significant associations were found between patients diagnosed with Stage II, Stage III or Stage IV cancer compared with patients diagnosed with stage I cancer (Tables 1, 2, and 3). Besides, gender, marital status, cancer type and duration of cancer were not significantly associated with the preferences for information, deliberation and decisional control by univariate analyses (*p* > .05) (Tables 1, 2 and 3).

**Table 2:** Association between sociodemographic and clinical characteristics and deliberation preference (*n* = 328)

	Low deliberation preference (*n* = 86)	High deliberation preference (*n* = 242)	Univariate analysis		Multivariate analysis	
Characteristics	(mean ± *SD*) or median (P_25_, P_75_) or *n* (%)	(mean ± *SD*) or median (P_25_, P_75_) or *n* (%)	Crude *OR* (95% CI)	*p* value	Adjusted *OR* (95% *CI*)	*p* value
Age (year)	56.99 ± 13.10	46.63 ± 13.15	0.938 (0.918–0.959)	**<.001**	0.942 (0.913–0.972)	**<.001**
Gender						
Female	42 (12.8)	116 (35.4)	Ref			
Male	44 (13.4)	126 (38.4)	1.037 (0.634–1.696)	.885		
Marital status				.117		
Unmarried	5 (1.5)	25 (7.6)	Ref			
Married	73 (22.3)	207 (63.1)	0.567 (0.209–1.536)	.265		
Divorced or widowed	8 (2.4)	10 (3.0)	0.252 (0.066–0.851)	.105		
Education level				**<.001**		**<.001**
Primary school	56 (17.1)	36 (11.0)	Ref		Ref	
Middle school	26 (7.9)	101 (30.8)	6.043 (3.313–11.021)	**<.001**	4.233 (2.170–8.219)	**<.001**
High school or college	2 (0.6)	63 (19.2)	32.667 (7.443–143.362)	**<.001**	20.606 (4.300–98.739)	**<.001**
Bachelor or above	2 (0.6)	42 (12.8)	49.000 (11.281–212.831)	**<.001**	33.290 (7.282–152.195)	**<.001**
Residence						
Rural	55 (16.8)	99 (30.2)	Ref		Ref	
Town or city	31 (9.5)	143 (43.5)	2.563 (1.540–4.265)	**<.001**	0.827 (0.404–1.692)	.603
Annual family income			‐	**.001**		.779
<50,000 RMB	55 (16.8)	99 (30.2)	Ref		Ref	
50,000–150,000 RMB	27 (8.2)	116 (35.4)	2.387 (1.401–4.067)	**.001**	1.139 (0.565–2.293)	.716
>150,000RMB	4 (1.2)	27 (8.2)	3.750 (1.248–11.272)	**.019**	1.585 (0.424–5.922)	.793
Cancer type				.115		
Breast	33 (10.1)	86 (26.2)	Ref			
Colorectal	16 (4.9)	25 (7.6)	0.600 (0.285–1.263)	.178		
Lung	37 (11.3)	131 (39.9)	1.359 (0.790–2.337)	.268		
Cancer stage				.125		
Stage I	3 (0.9)	7 (2.1)	Ref	**<.001**		**.048**
Stage II	4 (1.2)	43 (13.1)	4.607 (0.844–25.137)	.078	1.920 (0.287–12.834)	.501
Stage III	29 (8.8)	114 (34.8)	1.685 (0.410–6.919)	.469	1.009 (0.209–4.860)	.991
Stage IV	50 (15.2)	78 (23.8)	0.669 (0.165–2.707)	.573	0.482 (0.100–2.331)	.364
Duration of cancer(month)	3.5 (2.6)	4 (2.9.25)	0.999 (0.983–1.015)	.888		

*Note*: Bold *p* values are statistically significant. (P_25_, P_75_) is the interquartile range.

Abbreviations: 95% CI, 95% confidence interval; OR, odds ratio.

**Table 3:** Association between sociodemographic and clinical characteristics and decisional control preference (*n* = 328)

	Low decisional control preference (*n* = 206)	High decisional control preference (i = 122)	Univariate analysis		Multivariate analysis	
Characteristics	(mean ± *SD*) or median (P_25_, P_75_) or *n* (%)	(mean ± *SD*) or Median (P_25_, P_75_) or *n* (%)	Crude *OR* (95% *CI*)	*p* value	Adjusted *OR* (95% *CI*)	*p* value
Age (year)	53.05 ± 12.98	43.10 ± 13.15	0.945 (0.928–0.963)	**<.001**	0.957 (0.938–0.977)	**<.001**
Gender						
Female	114 (34.8)	56 (17.1)	Ref			
Male	92 (28.0)	66 (20.1)	0.685 (0.437–1.074)	.109		
Marital status				.122		
Unmarried	13 (4.0)	17 (5.2)	Ref			
Married	180 (54.9)	100 (30.5)	0.425 (0.398–1.011)	.108		
Divorced or widowed	13 (4.0)	5 (1.5)	0.565 (0.219–1.546)	.127		
Education level			‐	**<.001**		**.003**
Primary school	79 (24.1)	13 (4.0)	Ref		Ref	
Middle school	73 (22.3)	54 (16.4)	4.051 (1.879–8.735)	**<.001**	1.807 (0.734–4.446)	.198
High school or college	39 (11.9)	26 (7.9)	4.495 (2.268–8.909)	**<.001**	2.914 (1.387–6.121)	**.005**
Bachelor or above	15 (4.6)	29 (8.8)	11.749 (4.992–27.652)	**<.001**	5.529 (2.027–15.082	**.001**
Residence						
Rural	108 (32.9)	46 (14.0)	Ref		Ref	
Town or city	98 (29.9)	76 (23.2)	1.821 (1.153–2.876)	**.010**	0.986 (0.545–1.783)	.962
Annual family income			‐	**.001**		**.014**
<50,000 RMB	109 (33.2)	45 (13.7)	Ref		Ref	
50,000–150,000 RMB	86 (26.2)	57 (17.4)	1.605 (0.991–2.601)	.054	1.235 (0.695–2.192)	.472
>150,000 RMB	11 (3.4)	20 (6.1)	4.404 (1.952–9.935)	**<.001**	4.068 (1.566–10.565)	**.004**
Cancer type				.870		
Breast	73 (22.3)	46 (14.0)	Ref			
Colorectal	27 (8.2)	14 (4.3)	0.823 (0.391–1.731)	.607		
Lung	106 (32.3)	62 (18.9)	0.928 (0.572–1.506)	.763		
Cancer stage				**.008**		**.293**
Stage I	7 (2.1)	3 (0.9)	Ref		Ref	
Stage II	20 (6.1)	27 (8.2)	3.150 (0.724–13.713)	.126	1.767 (0.340–9.189)	.499
Stage III	88 (26.8)	55 (16.8)	1.458 (0.362–5.877)	.596	1.040 (0.219–4.928)	.961
Stage IV	91 (27.7)	37 (11.3)	0.949 (0.233–3.868)	.941	0.799 (0.166–3.856)	.780
Duration of cancer (month)	4 (2.8)	4 (2.8.25)	0.999 (0.984–1.015)	.914		

*Note*: Bold *p* values are statistically significant.

Abbreviations: 95% CI, 95% confidence interval; OR, odds ratio.

Based on multivariate analysis, age and education level were significantly associated with the preferences for information and deliberation, respectively, while age, education level and annual family income were significantly associated with the preference for decisional control (Tables 1, 2, and 3). Younger patients were more likely to have high preferences for information, deliberation and decisional control. Patients with higher levels of education were more likely to have high preferences for information and deliberation. Patients with a high school qualification (odds ratio [OR] = 2.968, 95% confidence interval [CI] [1.396, 6.308], *p* = .005) and a bachelor's degree or postgraduate qualification (OR = 5.811, 95% CI [2.146, 15.736], *p* = .001) were significantly more likely to have a high preference for decisional control compared with those with primary school education. However, patients with a middle school qualification were not significantly more likely to have a high preference for decisional control compared with those with primary school education (*p* > .05), which was inconsistent with the result of univariate analysis. Furthermore, patients with a high annual family income (>150,000RMB) were 4.068 times more likely to have a high preference for decisional control compared with those with a low annual family income (<50,000 RMB) (OR = 4.068, 95% CI [1.566,10.565], *p* = .004). However, patients with a middle annual family income (50,000 RMB–150,000 RMB) were not significantly more likely to have a higher level of preference for decisional control compared to those with low annual family income (<50,000 RMB) (*p* > .05) (Table 3). Overall, while not significantly associated with preference for decisional control, cancer stage was found to be significantly associated with preferences for information and deliberation. Patients diagnosed with Stage II, Stage III or Stage IV cancer were not significantly more likely to express high preferences for information and deliberation when compared with patients diagnosed with Stage I cancer, as was found in the univariate analysis (Tables 1 and 2). None of the other clinical characteristics were found to be significantly associated with the preferences for information, deliberation and decisional control (Tables 1, 2, and 3).

## 4 DISCUSSION AND CONCLUSION

### 4.1 Discussion

This study showed that preferences of cancer patients for participation could vary during different stages of the decision‐making process. Nearly half of the patients reported inconsistent preferences for information, deliberation and decisional control, which means that patients who express a low preference for decisional control may expect a high preference for information or (and) deliberation. More attention should be paid to the fact that patients have different preferences in terms of different components of the decision‐making process, which could help to decrease the mismatch between the preferred and actual levels of participation of patients in decision‐making practice. Besides, over one‐third (35.1%) and about one‐quarter (22.6%) of the patients preferred being active (concurrence of high preferences for information, deliberation and decisional control) and passive (concurrence of low preferences for information, deliberation and decisional control) during the whole decision‐making process, respectively, which was similar to the results of a pooled analysis of studies using the CPS to evaluate the preferred roles in treatment decision‐making among cancer patients.^24^

In our study, almost three‐quarters of the cancer patients expressed high preferences for information and deliberation, and nearly two‐thirds reported a low preference for decisional control. This was consistent with the findings of a qualitative study,^25^ which showed that most patients expressed a desire to participate in decision‐making with their doctors but also desired that the doctors make the treatment decision. In general, cancer patients require detailed information about their cancer and its treatment, and they desire information exchange.^26^, ^27^ Meeting information needs of the patients can yield several benefits, including increased patient satisfaction in decision‐making,^28^ reduced mood disturbances^29^ and better psychological well‐being.^30^ On the one hand, the medical staff should provide patients with their preferred information and provide more opportunities for patients to participate in the discussion of treatment options, so as to help them make the best treatment decision. On the other hand, we should respect the wishes of those who prefer their doctors to retain decisional control.

Our findings suggest that the majority of patients wanted as much information as possible, good or bad, and patient preference for information varies according to their age and education level. Younger patients were significantly more likely to express a stronger desire for information and patients with a higher level of education were more likely to have a high preference for information, which was consistent with the findings obtained from non‐Western ethnic minority cancer patients, as reported in a systematic review.^31^ In our study, patients living in a town or a city and patients with higher annual family incomes were significantly more likely to have a high preference for information as shown by univariate analysis. However, no significant associations were found by multivariate analysis, which may be due to the influence of confounding factors. Gender and marital status were not found to be significantly associated with the preference for information in our study, while a previous study found that women and married patients expressed significantly higher information needs,^32^ which needs further investigation. As for the clinical variables, cancer stage was found to be significantly associated with the preference for information, but no significant results were found on comparing patients diagnosed with Stage I cancer with patients diagnosed with Stage II, Stage III or Stage IV cancer, respectively, which needs further exploration, as a previous study showed no association between cancer stage and information preference,^32^ while a review showed that severity of illness was linked to the desire for information, with the most seriously ill patients showing less desire for information.^33^

Our findings related to the preference for deliberation were very similar to those of the preference for information. Most patients reported a high preference for discussing the treatment options with their doctors. Age and education level could be used to explain the variation in the preference for deliberation. Younger patients were significantly more likely to express a stronger desire to express and discuss treatment preferences than older patients. This may be related to the influence of family members of the patients. In China, family members play an important role in communicating with doctors, especially for patients with adult children. The older the patients, the more dependent they are on their family members. Patients with higher levels of education were more likely to express a high preference for deliberation. This may be explained by the fact that educated patients are better able to communicate with their doctors, as communication is essential and communication ability is important during the deliberation stage. The different results related to the association between residence, annual family income and the preference for deliberation by univariate and multivariate analyses may be due to the influence of confounding factors. More research should be conducted to explore the influencing factors of patients' preference for deliberation since no other related research has been retrieved currently.

As for the preference of decisional control, our findings showed that most patients reported a low preference for decisional control. Traditionally, Chinese patients are used to the paternalistic approach of their doctors and have a high degree of trust in their doctors. Most patients preferred delegating decisions to their doctors. This is similar to the findings obtained from Indian cancer patients.^34^ However, this is in stark contrast to the studies performed in the United States and in other developed countries, where a significant number of patients preferred a desire for decisional control.^24^, ^35^ Age, education level and annual family income could be used to explain the variation in the preference for decisional control. Older patients were significantly less likely to express a desire for personal control over the decisions. This is similar to the findings obtained by Sio et al.,^36^ which showed that older patients were more likely to delegate decision‐making to their providers. Patients with lower education levels in our sample were more likely to have a low preference for personal control over the decisions. These results are very similar to those reported by other studies that showed that patients with lower levels of education may feel less confident about becoming involved in decision‐making practices and more often expect a more passive role in medical decision‐making.^33^, ^35^, ^37^ In our study, patients with higher levels of annual family income were more likely to expect a high preference of personal control over the decisions. This may be due to the family‐centred nature of the Chinese culture. Patients who were financially well off worried less about the medical burden of treatment on their families and they wanted more personal control over their treatment decisions, which is similar to the result of a previous study.^38^ The other sociodemographic and clinical variables like gender, marital status, cancer type and duration of illness did not play a significant role in decisional control in our survey. These results are consistent with those of previous research,^34^ although other studies found a relationship.^33^, ^39^

As far as we are aware, our study preliminarily explored patient preferences for involvement in different stages of medical decision‐making and provided new insights into decision‐making practices from a Chinese perspective. However, some limitations should be kept in mind when interpreting our results. First, this research was carried out in a developed city, which could limit the generalization of our findings. Second, we could not rule out potential selection bias because of convenience sampling. Third, most data were self‐reported, with a risk of response bias. Fourth, the PEPMDS is a Chinese version scale, developed in the culture context of China, and its use limits the comparison of the findings with those of previous research.

### 4.2 Conclusion

Our results confirm that not everyone desires to be involved in the same way during treatment decision‐making practices and even the same patient desires to be involved at different levels during different stages of decision‐making. Most patients desired a high preference for information and/or discussion of treatment options with their doctors, but a low preference for personal control over the decisions. The sociodemographic characteristics like age, education level and economic status could be used to predict preferences of patients for participation in medical decision‐making practices. The clinical factors play a relatively small role in predicting patient preferences for all stages of the decision‐making process, which should be explored further.

### 4.3 Practice implications

As a practical implication of our results, healthcare providers should tailor the involvement approaches of patients by considering their preferences for information, deliberation and decisional control, to decrease the mismatch between the preferred and actual levels of involvement of the patients and improve patient satisfaction with decision‐making practices. Existing strategies focused on increasing the quality of treatment decisions for cancer patients need to deal with the preferences of patients for involvement, particularly for specific cancer populations, such as younger patients, those with higher levels of education and those with better economic conditions. To strive for the development of patient‐centred care, we should assess the preferences of individual patients and respond to their needs and preferences when possible.

## CONFLICT OF INTERESTS

The authors declare that there are no conflict of interests. 

## AUTHOR CONTRIBUTIONS

Lili Zhang and Yawei Liu contributed to the study design, research setting contact and part of the data acquisition. They contributed equally as corresponding authors. Lin Xiao contributed to the statistical analysis and drafted the manuscript with input from all authors. Meifang Peng contributed greatly to data collection. All authors contributed to the interpretation of the results and approved the final manuscript.

## Data Availability

The data are available from the corresponding authors and the first author.
